# Integration of Mn-ZnFe_2_O_4_ with S-g-C_3_N_4_ for Boosting Spatial Charge Generation and Separation as an Efficient Photocatalyst

**DOI:** 10.3390/molecules27206925

**Published:** 2022-10-15

**Authors:** Mohsin Javed, Waleed Bin Khalid, Shahid Iqbal, Muhammad Azam Qamar, Hamad Alrbyawi, Nasser S. Awwad, Hala A. Ibrahium, Murefah Mana Al-Anazy, Eslam B. Elkaeed, Rami Adel Pashameah, Eman Alzahrani, Abd-ElAziem Farouk

**Affiliations:** 1 Department of Chemistry, School of Science, University of Management and Technology, Lahore 54770, Pakistan; 2 Department of Chemistry, School of Natural Sciences (SNS), National University of Science and Technology (NUST), H-12, Islamabad 46000, Pakistan; 3 Pharmaceutics and Pharmaceutical Technology Department, College of Pharmacy, Taibah University, Medina 42353, Saudi Arabia; 4 Chemistry Department, Faculty of Science, King Khalid University, P.O. Box 9004, Abha 61413, Saudi Arabia; 5 Biology Department, Faculty of Science, King Khalid University, P.O. Box 9004, Abha 61413, Saudi Arabia; 6 Department of Semi Pilot Plant, Nuclear Materials Authority, El Maadi P.O. Box 530, Egypt; 7 Department of Chemistry, College of Science, Princess Nourah bint Abdulrahman University, P.O. Box 84428, Riyadh 11671, Saudi Arabia; 8 Department of Pharmaceutical Sciences, College of Pharmacy, AlMaarefa University, Riyadh 13713, Saudi Arabia; 9 Department of Chemistry, Faculty of Applied Science, Umm Al-Qura University, Makkah 24230, Saudi Arabia; 10 Department of Chemistry, College of Science, Taif University, P.O. Box 11099, Taif 21944, Saudi Arabia; 11 Department of Biotechnology College of Science, Taif University, P.O. Box 11099, Taif 21944, Saudi Arabia

**Keywords:** integration, charge separation, degrading organic pollutants, manganese, hydrothermal technique

## Abstract

The disposal of dyes and organic matter into water bodies has become a significant source of pollution, posing health risks to humans worldwide. With rising water demands and dwindling supplies, these harmful compounds must be isolated from wastewater and kept out of the aquatic environment. In the research presented here, hydrothermal synthesis of manganese-doped zinc ferrites’ (Mn-ZnFe_2_O_4_) nanoparticles (NPs) and their nanocomposites (NCs) with sulfur-doped graphitic carbon nitride (Mn-ZnFe_2_O_4_/S-g-C_3_N_4_) are described. The samples’ morphological, structural, and bonding features were investigated using SEM, XRD, and FTIR techniques. A two-phase photocatalytic degradation study of (0.5, 1, 3, 5, 7, 9, and 11 wt.%) Mn-doped ZnFe_2_O_4_ NPs and Mn-ZnFe_2_O_4_/(10, 30, 50, 60, and 70 wt.%) S-g-C_3_N_4_ NCs against MB was carried out to find the photocatalyst with maximum efficiency. The 9% Mn-ZnFe_2_O_4_ NPs and Mn-ZnFe_2_O_4_/50% S-g-C_3_N_4_ NCs exhibited the best photocatalyst efficiency in phase one and phased two, respectively. The enhanced photocatalytic activity of the Mn-ZnFe_2_O_4_/50% S-g-C_3_N_4_ NCs could be attributed to synergistic interactions at the Mn-ZnFe_2_O_4_/50% S-g-C_3_N_4_ NCs interface that resulted in a more effective transfer and separation of photo-induced charges. Therefore, it is efficient, affordable, and ecologically secure to modify ZnFe_2_O_4_ by doping with Mn and homogenizing with S-g-C_3_N_4_. As a result, our current research suggests that the synthetic ternary hybrid Mn-ZnFe_2_O_4_/50% S-g-C_3_N_4_ NCs may be an effective photocatalytic system for degrading organic pollutants from wastewater.

## 1. Introduction

Water contamination has attracted much attention from researchers since dye emissions from various sectors pose dangers to the public’s health and environment. Organic compounds, dissolved and suspended particles, and heavy metals are present in the complex effluents produced by the printing and textile dyeing industries [1]. About 15–50% of the azo dyes used during the dyeing process do not attach to the fabric and are washed away with the wastewater. This effluent is employed in irrigation processes, although it is terrible for crop growth and germination. Methylene blue (MB) is a basic cationic dye used in different sectors. It makes people more prone to cyanosis, tissue necrosis, shock, jaundice, vomiting, and a faster heartbeat. [2,3]. The wastewater must, therefore, unquestionably be cleaned of these colors. To treat contaminated effluents, a variety of chemical, biological, and physical methods have been used [4]. One of the green technologies for the treatment of industrial wastewater is the photocatalytic destruction of organic contaminants. Due to its incredible effectiveness and low cost, scientists have shown that photocatalytic decomposition is a suitable alternative strategy for the effective decomposition of pollutants [5,6]. The employment of alternative techniques to eliminate by-products is not required in photocatalysis procedures. Numerous nanostructured semiconductor photocatalysts have been explored, including TiO_2_, ZnO, CuO, NiS, SnO_2_, ZrO_2_, and WO_3_. Due to their significant band gaps and quick electron-hole recombination, these photocatalysts have limitations in visible-light photocatalysis [7,8].

Therefore, creating innovative visible-light-induced photocatalysts with higher activity has been a hot topic for a long time. Due to the superior cation and anion adsorption abilities, low band gap energy, and reduced electron-hole recombination, ferrite (Fe_2_O_4_) nanoparticles have particular relevance among photocatalysts [9]. These ferrite NPs have prospective applications in wastewater treatment, biomedicine, electrical devices, energy storage, EMR shielding, and the recording medium. They have a band gap of about 1.9 eV and are highly stable. They are also less harmful and inexpensive, have strong electronic conduction, are recyclable, and are environmentally benign. Their photocatalytic activity is unfortunately constrained for practical use by low quantum efficiency [9,10,11]. 

Many studies have shown that doping ZnFe_2_O_4_ with appropriate metal ions and combining it with a suitable semiconductor material improve optical and photocatalytic characteristics. Patil et al. used the co-precipitation approach to manufacture Gd3+-doped ZnFe_2_O_4_ nanoparticles, demonstrating enhanced MB degradation of roughly 99% compared to pure ZnFe_2_O_4_ (95% degradation in 240 min.) [12]. According to Ajithkumar et al., yttrium-doped zinc ferrite made by solution combustion showed 95% MB degradation in 180 min. The photocatalytic efficiency of Y-doped ZnFe_2_O_4_ is greater than that of pure zinc ferrite [13]. Compared to ZnFe_2_O_4_, cobalt-doped zinc ferrite more effectively oxidized methylene blue under visible light. Numerous studies have found that ZnFe_2_O_4_ has a finite band gap energy and, as a result, may combine with S-g-C_3_N_4_ to create an efficient heterojunction [14]. Similarly, Savunthari et al. constructed (Cu, Bi) codoped ZnFe_2_O_4_ nanoparticles via the solution combustion method. The codoped NPs showed an enhanced degradation of bisphenol A compared to undoped NPs [15].

The g-C_3_N_4_ semiconductor has demonstrated remarkable photocatalytic competency under visible light due to its advantageous traits, such as excellent stability and a lowered band gap energy that boosts its capacity to absorb visible radiations [16,17]. However, the rapid recombination of photoinduced e^−^/h^+^ pairs in g-C_3_N_4_ makes it inappropriate for use as a photocatalyst [18]. Consequently, many attempts have been undertaken to overcome this constraint, including vacancy, heterojunction creation, and combining the g-C_3_N_4_ with other metal oxides and nonmetals such as sulphur [19,20,21,22]. S-doping modifies the band gap of g-C_3_N_4_ and improves the mobility and separation of the e-h pairs by stacking its 2p orbitals on the VB of bulk g-C_3_N_4_ [23]. A simple molten salt approach was successfully used by Keke et al. to produce sulfur-doped g-C_3_N_4_. The photocatalytic performance of S-doped g-C_3_N_4_ toward methylene blue and tetracycline was 10 and 20 times that of bulk g-C_3_N_4_ [24]. Similarly, Xin et al. successfully synthesized highly active S-doped g-C_3_N_4_ by employing thiourea and melamine as the precursors. The synthesized S-doped g-C_3_N_4_ exhibited remarkable photocatalytic efficacy against rhodamine B (RhB) [25]. Basaleh used soft and hard templates to create ZnFe_2_O_4_/S-g-C_3_N_4_. The photocatalytic efficiency of ZnFe_2_O_4_/S-g-C_3_N_4_ against acridine orange was 4.4 and 6.3 fold that of ZnFe_2_O_4_ and bulk g-C_3_N_4_, respectively [26]. 

Thus, owing to the improved charge separation abilities, it is suggested to produce a metal-ZnFe_2_O_4_/S-g-C_3_N_4_ heterojunction to realize a significant photocatalytic performance. In this study, hybrid Mn-ZnFe_2_O_4_/S-g-C_3_N_4_ nanocomposites were synthesized successfully via a surfactant (PEG)-assisted hydrothermal process, and its efficiency for removing MB under sunlight was investigated. In step one, the manganese-doped zinc ferrite (Mn-ZnFe_2_O_4_) nanoparticles were synthesized with varying chromium percentages (0.5, 1, 3, 5, 7, 9, and 11 wt.%). The effect of Mn^2+^ substitution on the photocatalytic properties of zinc ferrite was observed. The 9% Mn-ZnFe_2_O_4_ sample manifested the best absorption of solar light and degradation efficiency. In step two, the 9% Mn-ZnFe_2_O_4_ nanoparticles were homogenized with diverse concentrations of S-g-C_3_N_4_ (10, 30, 50, and 70 wt.%) to produce Mn-ZnFe_2_O_4_/S-g-C_3_N_4_ with enhanced photocatalytic activity. The 9% Mn-ZnFe_2_O_4_/50% S-g-C_3_N_4_ nanocomposite executed the best photocatalytic activity compared to pure ZnFe_2_O_4_, 9% Mn-ZnFe_2_O_4,_ and S-g-C_3_N_4_. The results depicted that the enhanced photocatalytic activity of the 9% Mn-ZnFe_2_O_4_/50% S-g-C_3_N_4_ nanocomposite was because of the improved absorption of sunlight and better separation of e^-^/h^+^ pairs between Mn-ZnFe_2_O_4_ and S-g-C_3_N_4_. To our knowledge, the synthesis of Mn-ZnFe_2_O_4_/S-g-C_3_N_4_ heterojunctions via the hydrothermal approach has never been used.

## 2. Experimental

### 2.1. Chemicals

Thiourea (CH_4_N_2_S), polyvinyl pyrrolidone, methylene blue (C_16_H_18_ClN_3_S), zinc sulphate heptahydrate (ZnSO_4_·7H_2_O), iron (III) chloride anhydrous (FeCl_3_), manganese (II) chloride (MnCl_2_), and sodium sydroxide (NaOH) were acquired from Merck and used. 

### 2.2. Synthesis of Chromium-Doped Zinc Ferrites

A series of Mn-doped zinc ferrites (Mn-ZnFe_2_O_4_) with varying manganese percentages (0.5, 1, 3, 5, 7, and 9 wt.%) were produced using a surfactant-assisted hydrothermal method [6]. For the preparation of 0.5% Mn-ZnFe_2_O_4_, three solutions, A, B, and C, were made preceding the synthesis. The solutions, A, B, and C, were made by dispersing 0.010 g of MnCl_2_·4H_2_O, 1.865 g of ZnCl_2_·7H_2_O, and 2.435 g of FeCl_3_ in 50 mL of deionized water. Then, these solutions (A, B, and C) were intermixed, and 10 mL of PVP was added as a surfactant to prevent the aggregation of nanoparticles. The pH of the solution was adjusted to 11 by adding NaOH; next, the suspension was loaded to a Teflon-lined autoclave. The autoclave was heated to 175 °C for 10 h and then cooled to ambient temperature. The resultant precipitates were separated by filtering, rinsed with deionized water and pure ethanol, and then dried at 85 °C in an oven. Other percentages of Mn-ZnFe_2_O_4_ (0, 1, 3, 5, 7, and 9 wt.%) were also synthesized using the same method [27].

### 2.3. Synthesis of S-g-C_3_N_4_

Thiourea was heated to 560 °C for 6 h at a rate of 5 °C per minute in a muffle furnace to form S-g-C_3_N_4_. After cooling to ambient temperature, the resulting yellowish S-g-C_3_N_4_ was stored [17,28]. 

### 2.4. Synthesis of Mn-ZnFe_2_O_4_/S-g-C_3_N_4_

**Scheme 1:**
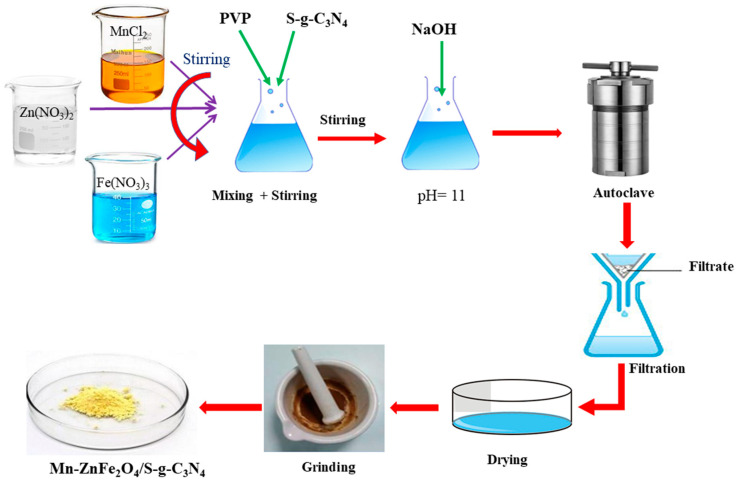
Schematic depiction for the production of Mn-ZnFe_2_O_4_/S-g-C_3_N_4_.

Using a surfactant-assisted hydrothermal technique, 9% Mn-ZnFe_2_O_4_ was combined with various amounts of S-g-C_3_N_4_ (10, 30, 50, 60, and 70 wt.%) to produce a range of Mn-ZnFe_2_O_4_/S-g-C_3_N_4_ nanocomposites. For the preparation of 9% Mn-ZnFe_2_O_4_/10%S-g-C_3_N_4_, firstly, four solutions, A, B, C, and D, were made by dispersing 0.185 g of manganese chloride in 50 mL of water (Solution A), 1.706 g of zinc chloride in 50 mL of water (Solution B), 2.435 g of FeCl_3_ in 50 mL of water (Solution C), and 0.18 g of S-g-C_3_N_4_ in 50 mL of water (Solution D). Then, three solutions, A, B, and C, were added to solution D and homogenized for 1 h along with the addition of 10 mL of PVP as a surfactant. The following steps were the same as for the synthesis of Mn-ZnFe_2_O_4_ NPs. Moreover, the same process was repeated to synthesize the 9% Mn-ZnFe_2_O_4_/S-g-C_3_N_4_ containing 30, 50, 60, and 70 wt.% of S-g-C_3_N_4_. The schematic diagram (Scheme 1) depicts the synthesis procedure for Mn-ZnFe_2_O_4_/S-g-C_3_N_4_ NCs, and Table 1 lists the precise composition. 

### 2.5. Photocatalytic Activity

The photocatalytic activity of zinc ferrites, Mn-doped zinc ferrites, sulphur-doped graphitic carbon nitride, and nanocomposites was studied under solar light irradiation. The aqueous solution of an organic dye, methylene blue, was used as the standard contaminant. Then, 0.2 g of each photocatalyst was added to the beaker in which 100 mL of MB solution (10 mg L^−1^) was added and allowed to stir in the dark for about 45 min to attain the adsorption–desorption equilibrium. After that, the suspension was positioned under solar light in an open atmosphere, and aliquots of 5 mL were collected after every 30 min. A UV-Vis spectrophotometer was used to evaluate the photocatalytic activity of the collected samples after centrifugation [29].

### 2.6. Characterization

The structure of the synthesized catalysts was determined by applying XRD (Bruker AXS, D8-S4, Madison WI, USA) using Cu Kα radiation (k = 1.54056 Å) at 40 kV and 30 mA at room temperature, whereas elemental content and morphology were found using SEM-EDS (Hitachi, S-4800, Tokyo, Japan). The UV-visible and photocatalytic absorption spectra were measured using a UV-Vis-NIR spectrophotometer (UV-770, Jasco, Tokyo, Japan) from 800 nm to 200 nm wavelengths. Using a transmission electron microscope, the surface morphologies of the photocatalysts were examined (TEM, JEOL-JEM-1230, Peabody, MA, USA). FTIR spectrometers measured functional groups in the 4000–400 cm^−1^ range with a resolution of 1 cm^−1^ (Perkin 400 FTIR, Waltham, MA, USA). 

## 3. Results and Discussion

### 3.1. TEM and EDX Analyses

**Figure 1:**
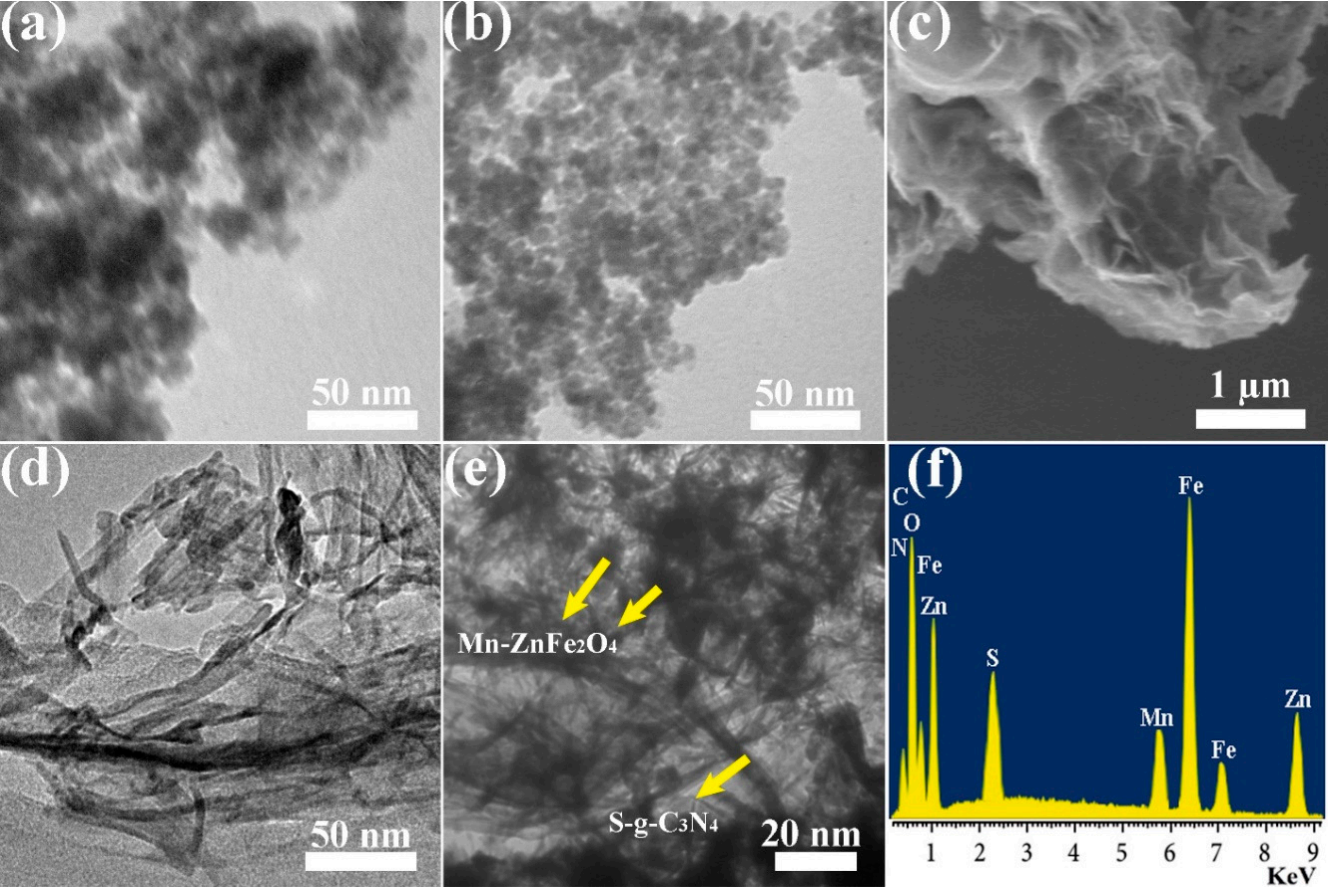
TEM profiles of (**a**) ZnFe_2_O_4_, (**b**) 9% Mn-ZnFe_2_O_4_, (**c**) SEM profile of S-g-C_3_N_4_, (**d**) TEM image of S-g-C_3_N_4,_ and (**e**) 9% Mn-ZnFe_2_O_4_/50% S-g-C_3_N_4_ NCs. (**f**) EDX of 9% Mn-ZnFe_2_O_4_/50% S-g-C_3_N_4_ NCs.

The size, shape, and distribution of the particles and the elemental components of the as-prepared materials were disclosed by TEM, SEM, and EDX spectra. Due to their large surface area due to their nanoscale size and solid magnetic interaction, undoped ZnFe_2_O_4_ and Mn-doped ZnFe_2_O_4_ nanoparticles were observed to be crystalline and included agglomerated, irregular, spherical nanoparticles with an average size of 20–38 nm (Figure 1a,b). The proper exposure to different hosts made possible by the efficient synthesis of Mn-ZnFe_2_O_4_ at the nanoscale also guaranteed the single-domain character of the particles for the increase in magnetic remanence and decrease in magnetic coercivity. The S-g-C_3_N_4_ specimen had some creases and a beehive-like shape (Figure 1c,d). This supramolecular complex was synthesized using a mixture of three different precursors, and the pyrolysis of the complex resulted in the formation of wrinkles. These wrinkles facilitated high interfacial contact between S-g-C_3_N_4_ and other components, which improved the transfer and separation of charge carriers in the NCs. The 9% Mn-ZnFe_2_O_4_/50% S-g-C_3_N_4_ NCs’ TEM pictures showed that several Mn-ZnFe_2_O_4_ nanoparticles seemed to be incorporated in the S-g-C_3_N_4_ matrix. As shown in Figure 1e, it was clear that S-g-C_3_N_4_ had firmly encircled several Mn-ZnFe_2_O_4_ NPs. The 9% Mn-ZnFe_2_O_4_/50% S-g-C_3_N_4_ NCs had sheet-like clusters with O, Zn, Fe, C, N, and Mn as their primary components, according to EDX mapping in Figure 1f, for the 9% Mn-ZnFe_2_O_4_/50 % S-g-C_3_N_4_ binary hybrid photocatalyst.

### 3.2. FTIR Analysis

**Figure 2:**
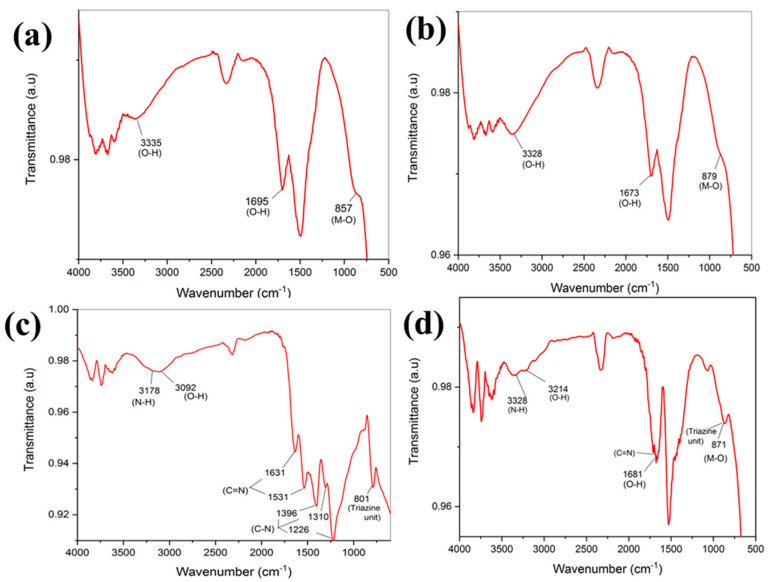
FTIR spectrum of composites of ZnFe_2_O_4_ (**a**), S-g-C_3_N_4_ (**b**), 9% Mn-ZnFe_2_O_4_ (**c**), and 9% Mn-ZnFe_2_O_4_/50S-gC_3_N_4_ (**d**).

In the wave number range of 4000–450 cm^−1^, the FTIR spectra of the produced photocatalysts were compared (Figure 2). The observed FTIR spectrum of ZnFe_2_O_4_ confirmed the presence of the M-O bond at 879 cm^−1^ and the absence of all functional groups. The peak at 3335 cm^−1^ was due to the O-H bond stretching while the peak at 1696 cm^−1^ was due to the bending of the O-H bond (Figure 2a) [5]. When the FTIR spectrum of 9% Mn-ZnFe_2_O_4_ was compared to pure ZnFe_2_O_4_, there was little difference in the peak positions. The effective production of Mn-doped ZnFe_2_O_4_ was confirmed by the modest shift in peak positions (Figure 2b) [30,31]. The FTIR spectrum of S-g-C_3_N_4_ exhibited a broad band at 3178 cm^−1^ due to O-H bond stretching; the peaks between 1500 and 2000 cm^−1^ were due to the stretching vibrations of C=N, and peaks between 1500 and 1000 cm^−1^ were due to C-N bond stretching, as shown in Figure 2c [32]. A peak at 805 cm^−1^ indicated the triazine unit. The FTIR spectrum of the composite contained the peaks corresponding to both Mn-ZnFe_2_O_4_ and S-gC_3_N_4,_ signifying that the Mn-ZnFe_2_O_4_/S-g-C_3_N_4_ composite was formed successfully (Figure 2d) [33,34,35,36].

### 3.3. XRD Analysis

**Figure 3:**
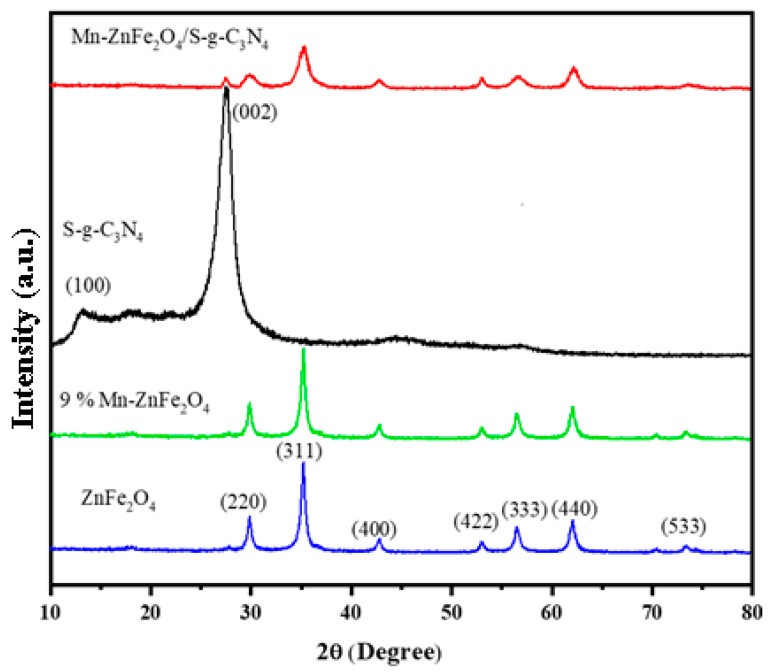
XRD spectrum of ZnFe_2_O_4_, 9% Mn-ZnFe_2_O_4_, S-g-C_3_N_4,_ and 9% Mn-ZnFe_2_O_4_/50% S-g-C_3_N_4_.

The X-ray diffractogram of the samples of ZnFe_2_O_4_, 9% Mn-ZnFe_2_O_4_, S-g-C_3_N_4,_ and 9% Mn-ZnFe_2_O_4_/50% S-g-C_3_N_4_ is shown in Figure 3. In the XRD spectra of pure ZnFe_2_O_4,_ seven peaks were observed with crystal facets (220), (311), (400), (422), (511), (440), and (533) at 2θ = 30°, 34.8°, 42.6°, 53°, 56°, 61.8°, and 73.2°, which fitted well with the pattern of standard zinc ferrites with JCPDS file 22-1012 [12,30,31,37,38]. The distinctive peaks of zinc ferrites in the XRD spectra of Mn-ZnFe_2_O_4_ showed that the structure of zinc ferrite does not significantly alter when Mn metal is doped into it. Two characteristic peaks were detected in the XRD pattern of S-g-C_3_N_4_; the crystal plane (002) was attributed to the interlayer assembling of aromatic systems, and the plane (100) was ascribed to the inter-planar arrangement of aromatic systems (JCPDS file # 00-087-1526) [39,40]. The crystal phase of Mn-ZnFe_2_O_4_ remained intact after coupling with S-g-C_3_N_4_, and the (002) crystal plane of S-g-C_3_N_4_ was indicated in the composite systems. In 9%Mn-ZnFe_2_O_4_/50% S-g-C_3_N_4_ composites, owing to the high crystallinity of Mn-ZnFe_2_O_4,_ the characteristic peaks of Mn-ZnFe_2_O_4_ were prominent. The emergence of the distinct peaks of Mn-ZnFe_2_O_4_ and S-g-C_3_N_4_ in composites demonstrated the successful production of Mn-ZnFe_2_O_4_/S-g-C_3_N_4_ composites [34,35,41,42]. 

### 3.4. Photocatalytic Degradation Study

**Figure 4:**
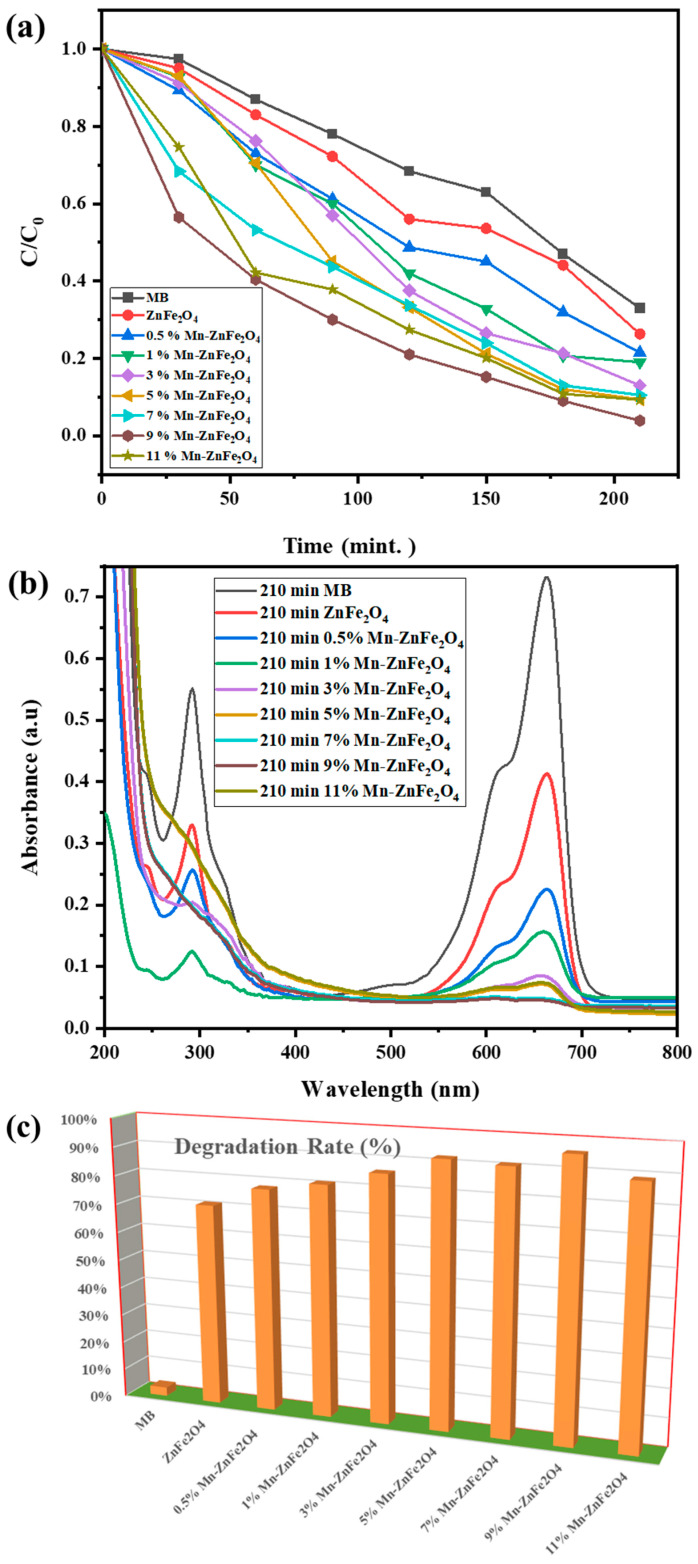
Degradation rate (**a**), degradation contours (**b**), and % degradation of MB (**c**) by Mn-ZnFe_2_O_4_ NPs.

**Figure 5:**
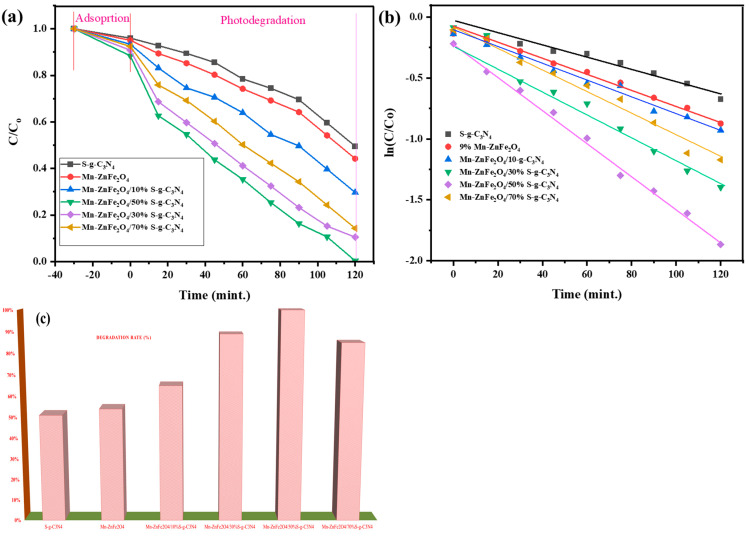
Photocatalytic degradation rate (**a**), % degradation (**b**), and kinetic characteristics of MB by Mn-ZnFe_2_O_4_/S-g-C_3_N_4_ NCs (**c**).

The photocatalytic activity of synthesized samples was detected in two phases. The photocatalytic activities of ZnFe_2_O_4_ and Mn-ZnFe_2_O_4_ NPs were first examined in the presence of sunlight using an aqueous methylene blue solution (Figure 4a). The rate of dye disintegration was monitored using a UV-Vis spectrophotometer with a wavelength range of 200–800 nm (Figure 4a). According to the degradation contours (Figure 4b) and percentile degradation graphs (Figure 4c), when we increased the Mn^+2^ doping (0.5 to 9 wt.%), there was a gradual improvement in the photocatalytic activity of Mn-doped zinc ferrite nanoparticles. Since the Mn^+2^ doping decreased the band gap of ZnFe_2_O_4_ and facilitated the enhanced generation of the e^−^/h^+^ pair, the photocatalytic efficiency of Mn-ZnFe_2_O_4_ was better than ZnFe_2_O_4_. It was observed that the insertion of various metal ions into the pure ferrite can affect its optical and structural properties, which can enhance the ferrite’s photocatalytic ability [43]. When the concentration of doped Mn^+2^ ions was increased beyond this point (<9 wt.%), the photocatalytic activity of Mn_x_Zn_1−x_Fe_2_O_4_ NPs was reduced (Figure 5a,b). This Mn (9 wt.%) is the optimum doping concentration for Mn-ZnFe_2_O_4_ NPs. The observed degradation efficiencies of Mn-ZnFe_2_O_4_ catalysts with different manganese concentrations (0, 0.5, 1, 3, 5, 7, and 9 wt.%) were 71%, 78%, 81%, 86%, 92%, 91%, 95%, and 89%, respectively, after 210 min of sunlight irradiation. Thus, the 9% Mn-ZnFe_2_O_4_ NPs exhibited the maximum photocatalytic efficiency compared to other nanoparticles (Figure 4c).

In the next phase, the Mn-ZnFe_2_O_4_/S-g-C_3_N_4_ NCs were produced by mixing 9% Mn-ZnFe_2_O_4_ NPs with diverse amounts of S-g-C_3_N_4_ (as given in Table 1. Then, the photocatalytic activity of the produced NCs was checked every 15 min. The samples were placed in the dark to establish adsorption–desorption equilibrium between the dye and the fabricated NCs before sunlight exposure, as described by Mudassar et al. [44]. According to Figure 5b, the photocatalysts absorbed modest amounts of MB. After that, the samples were exposed to sunlight, and the 9% Mn-ZnFe_2_O_4_/50S-g-C_3_N_4_ NCs exhibited the largest dye degradation relative to the other samples (Figure 5a). It was evident from the degradation contours (Figure 5a) and percent degradation plots (Figure 5b) that the dye degradation increased with an increasing S-g-C_3_N_4_ concentration in the Mn-ZnFe_2_O_4_/S-g-C_3_N_4_ NCs up to 50% and then dropped for ZnFe_2_O_4_/S-g-C_3_N_4_ NCs containing S-g-C_3_N_4_ contents >50%. After 120 min of exposure to sunlight, the measured degradation efficiencies of S-g-C_3_N_4_ and 9% Mn-ZnFe_2_O_4_/(0, 10, 30, 50, 60, and 70 wt.%) S-g-C_3_N_4_ NCs were 51%, 54%, 65%, 89%, 100%, and 85%, respectively. Better charge separation and transportation via Mn-ZnFe_2_O_4_ and S-g-C_3_N_4_ coupling, as well as improved visible light absorption due to Mn doping in ZnFe_2_O_4_, might be responsible for the enhanced photocatalytic efficiency of 9% Mn-ZnFe_2_O_4_/50% S-g-C_3_N_4_ NCs [41,42,45]. Figure 5b depicts the percentage of photocatalytic degradation of MB by the corresponding NCs.

The Langmuir–Hinshelwood model was applied to understand the kinetics of Mn-ZnFe_2_O_4_ (Supplementary Materials: Figure S1) and Mn-ZnFe_2_O_4_/S-g-C_3_N_4_ NCs, and Figure 5c shows the resultant graph [38]. Figure 5c exhibits that the dye degradation by the NCs under sunlight was fit to pseudo-first-order kinetics. The rate constant (k) values of Mn-ZnFe_2_O_4_/S-g-C_3_N_4_ NCs are summarized in Table 2. 

Therefore, the highest and lowest calculated “k” values were found for ZnFe_2_O_4_/50% S-g-C_3_N_4_ NCs (0.0142 min^−1^) and S-g-C_3_N_4_ (0.00515 min^−1^), respectively. The ZnFe_2_O_4_/50% S-g-C_3_N_4_ NCs completely decolorized the MB in 120 min, and its “k” value was 2.1 and 2.75 times more than that of ZnFe_2_O_4_ and S-g-C_3_N_4,_ respectively. The rate of dye degradation increased as the concentration of S-g-C_3_N_4_ increased from 10% to 50% in ZnFe_2_O_4_/50% S-g-C_3_N_4_ NCs but then declined for NCs with a greater concentration of S-g-C_3_N_4_ (<50%). Accordingly, the observed optimal concentration for ZnFe_2_O_4_/S-g-C_3_N_4_ NCs is 50% S-g-C_3_N_4_. A further rise in the concentration of S-g-C_3_N_4_ could result in the formation of e-h pair combination centers, which would gradually reduce the photocatalytic efficiency of NCs [32,44]. A preliminary study is required to conduct a more in-depth analysis of this justification. According to Table 3, the photocatalytic efficiency of ZnFe_2_O_4_/50% S-g-C_3_N_4_ NC was noticeably superior to that of several previously reported studies [46,47,48]. The better photocatalytic efficiency of the NC might be due to the development of good heterojunctions between ZnFe_2_O_4_ and S-g-C_3_N_4_ as compared to the previously reported composites. The Mn atoms may also facilitate the transportation and separation of the e^−^/h^+^ in the composite [21,28,29]. Because the ZnFe_2_O_4_/50% S-g-C_3_N_4_ NC was the most efficient photocatalyst, it was used in the recycling study.

**Figure 6:**
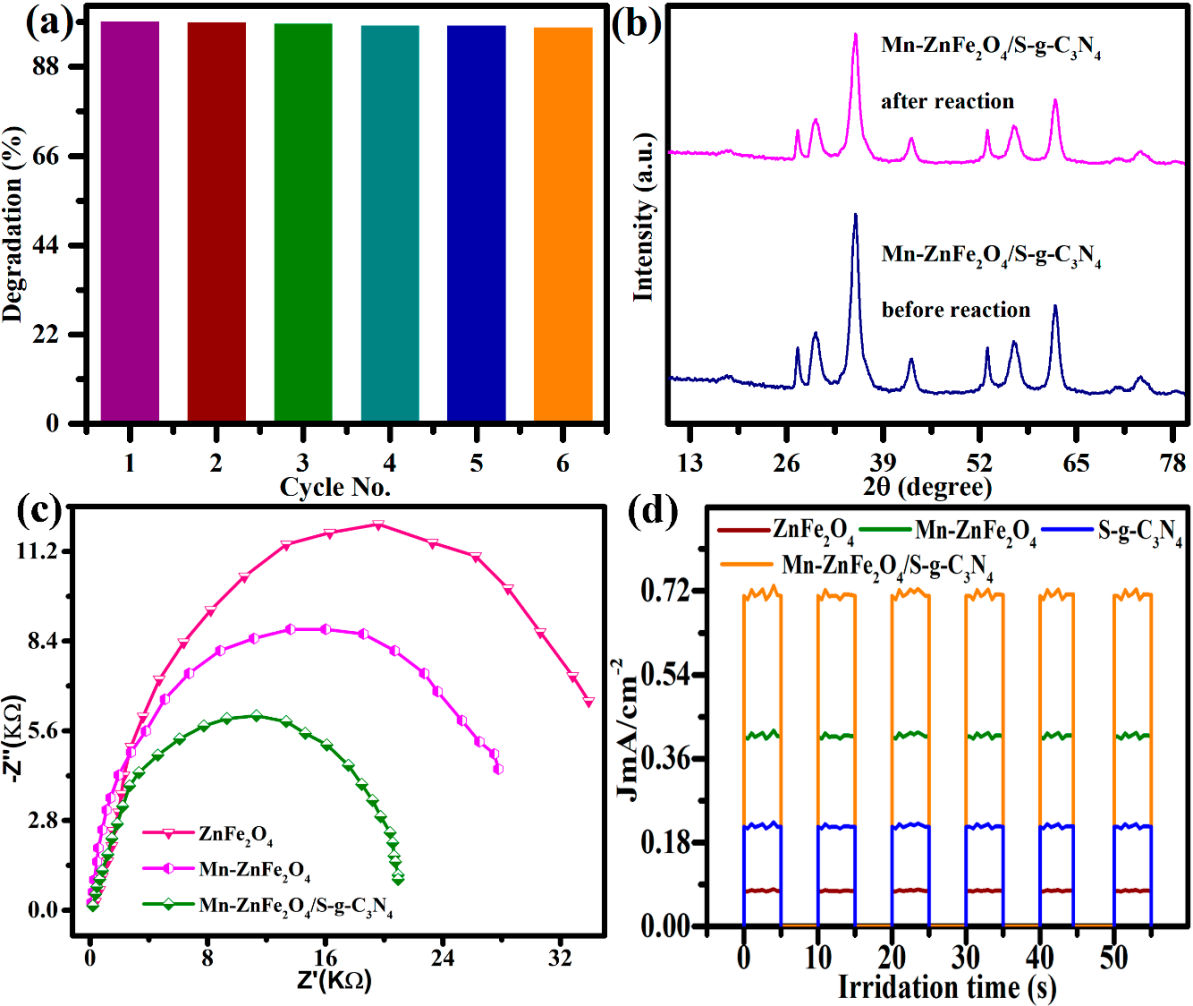
(**a**) Cyclic stability of the Mn-ZnFe_2_O_4_/50% S-g-C_3_N_4_ NCs through the sixth cycle. (**b**) The structural stability of Mn-ZnFe_2_O_4_/50% S-g-C_3_N_4_ NCs was determined by comparing XRD patterns obtained before the first cycle and after the sixth recycling experiment. (**c**) EIS Nyquist plots of ZnFe_2_O_4_, Mn-ZnFe_2_O_4,_ and Mn-ZnFe_2_O_4_/50% S-g-C_3_N_4_ NCs and (**d**) transient photocurrent responses of ZnFe_2_O_4_, S-g-C_3_N_4_, Mn-ZnFe_2_O_4,_ and Mn-ZnFe_2_O_4_/50% S-g-C_3_N_4_ NCs in visible-light irradiation.

The produced Mn-ZnFe_2_O_4_/50% S-g-C_3_N_4_ NCs are shown in Figure 6a throughout their stability cycles and photocatalyst reuse. The recycling and stability test was used to gauge how well the material as prepared performed in real-world applications. Six sequential cycles of MB dye degradation under direct sunshine irradiation were used to test the photocatalyst’s stability. After six cycles of the photocatalytic MB elimination test, Mn-ZnFe_2_O_4_/50% S-g-C_3_N_4_ NCs’ crystallographic alterations were also examined using XRD, and the relevant findings are shown in Figure 6b. The stability of the crystal structure of Mn-ZnFe_2_O_4_/50% S-g-C_3_N_4_ NCs is shown by the lack of noticeable change in the XRD curves before and after the photocatalytic test, as shown in Figure 6b.

The improvements in the Mn-ZnFe_2_O_4_/50% S-g-C_3_N_4_ photocatalyst’s photocatalytic MB removal ability were further examined using Figure 6c. The transient photocurrent responses of the ready photocatalysts are shown in Figure 6c. The law of transient photocurrent responses and the photocatalytic MB elimination efficiency of the various photocatalysts may be compared using the photocatalytic test results shown in Figure 6c. The photocurrent density of Mn-ZnFe_2_O_4_/50% S-g-C_3_N_4_ NCs is was highest, indicating the material’s best optical responsiveness and output capacity of photogenerated carriers during the photocatalytic reaction. ZnFe_2_O_4_, Mn-ZnFe_2_O_4_, and Mn-ZnFe_2_O_4_/50% S-g-C_3_N_4_ NCs’ EIS spectra are shown in Figure 6d. The outstanding photogenerated carrier migration and separation efficiency of Mn-ZnFe_2_O_4_/50% S-g-C_3_N_4_ NCs is shown by the fact that their EIS curve’s arc is noticeably lower than that of ZnFe_2_O_4_. Furthermore, the PL was measured using a 330 nm excitation wavelength to assess the charge transfer and separation effectiveness of ZnFe_2_O_4_, Mn-ZnFe_2_O_4,_ and Mn-ZnFe_2_O_4_/S-g-C_3_N_4_ heterojunctions, as shown in Figure S3.

### 3.5. Scavenging Activity

**Figure 7:**
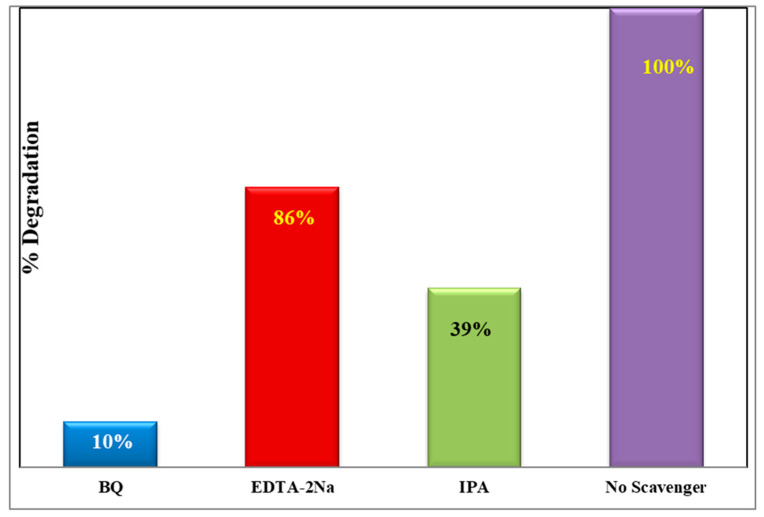
Role of radical scavengers in the photocatalytic degradation of MB with Mn-ZnFe_2_O_4_/50% S-g-C_3_N_4_.

The primary active species for the decomposition of organic pollutants in water are typically superoxide radicals (^•^O_2_), hydroxyl radicals (^•^OH), and photogenerated holes (h^+^) [46]. Therefore, the active species-generating capacity was examined by incorporating the appropriate scavengers into the suspensions of the MB degradation in the presence of the Mn-ZnFe_2_O_4_/50% S-g-C_3_N_4_ photocatalyst. In particular, isopropanol (IPA) was specifically utilized to trap ^•^OH, EDTA-2Na to trap holes (h^+^), and benzoquinone (BQ) to trap ^•^O_2_. After adding benzoquinone, the efficiency of MB degradation was lowered by 90%. IPA and EDTA-2Na, on the other hand, only suppressed the degradation rates of MB by 61% and 37%, respectively. The effect of trapping chemicals on the dye degradation reaction is depicted in Figure 7. The outcomes demonstrated that ^•^OH and •O^−2^ are the main reactive species involved in photocatalytic dye degradation rather than the holes (h^+^).

## 4. Photocatalytic Degradation Mechanism

**Figure 8:**
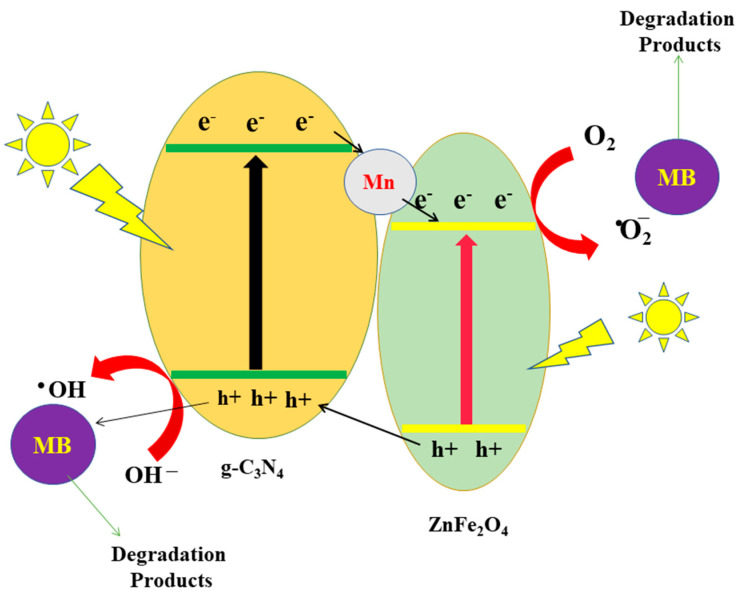
A schematic MB sunlight catalytic degradation mechanism over the Mn-ZnFe_2_O_4_/S-g-C_3_N_4_ NCs.

The accelerated degradation of methylene blue by photocatalysts may be attributed to the production of e^−^/h^+^ pairs in the synthesized photocatalysts as predicted via a schematic sketch (Figure 8). Both Mn-ZnFe_2_O_4_ and S-g-C_3_N_4_ were excited and e^−^/h^+^ pairs were produced on their respective conduction bands (CB) and valence bands (VB) when solar light was irradiated on Mn-ZnFe_2_O_4_/S-g-C_3_N_4_ [47]. Based on the CB/VB edge potentials, the photo-induced electrons easily migrated from the conduction band (CB) of Mn-ZnFe_2_O_4_ to the CB of S-g-C_3_N_4_ since the CB of Mn-ZnFe_2_O_4_ was lower in potential than that of S-g-C_3_N_4_. In addition, the holes that were created in the VB of S-g-C_3_N_4_ had the potential to migrate to Mn-ZnFe_2_O_4_ [48]. In the hybrid composite, the presence of Mn atoms not only lowered the value of Eg but also served as the facilitator for the transit of electrons from S-g-C_3_N_4_ to ZnFe_2_O_4_. Therefore, by increasing the separation of photoexcited e^−^/h^+^ pairs, doping could significantly lower the chance of charge recombination. The produced e^−^/h^+^ pairs combined with the oxygen and water molecules taken up on the surface of the photocatalyst to generate (^•^OH and ^•^O^−2^) the reactive oxygen species (ROS) [1]. Then, the generated radicals were consumed in the degradation of MB via an oxidative mechanism.

## 5. Conclusions

In summary, ZnFe_2_O_4_, Mn-ZnFe_2_O_4_ nanoparticles, and a series of Mn-ZnFe_2_O_4_/S-g-C_3_N_4_ nanocomposites were developed via a straightforward hydrothermal technique. XRD, TEM, EDX, and FTIR techniques were used to investigate the structure and purity of the samples. The degradation of MB at room temperature was carried out using ZnFe_2_O_4_, Mn-ZnFe_2_O_4_, and Mn-ZnFe_2_O_4_/S-g-C_3_N_4_. The synthesized samples were tested photocatalytically against MB, and it was discovered that the 9%Mn-ZnFe_2_O_4_/50% S-g-C_3_N_4_ had a very high catalytic efficiency. It was established through the radical scavenging experiment that the 9%Mn-ZnFe_2_O_4_/50% S-g-C_3_N_4_ utilized electrons, holes, and ROS for MB degradation. For six sequential catalytic cycles, the nanocomposites shown exceptional stability and continuously high levels of MB degradation. The separation and mobility of photoinduced e^−^/h^+^ pairs in 9%Mn-ZnFe_2_O_4_/50% S-g-C_3_N_4_ may be considerably enhanced by the fine interfaces produced and the synergistic effect between Mn and ZnO as supported by transient photocurrent responses. Both for NPs and NCs, it was found that a rate constant for the dye reduction reaction was pseudo-first order. As a result, the 9% Mn-ZnFe_2_O_4_/50% S-g-C_3_N_4_ heterojunction is a promising contender and may find use in the photocatalytic destruction of organic pollutants to purify water.

## Figures and Tables

**Table 1:** Composition of the synthesized Mn-ZnFe_2_O_4_/S-g-C_3_N_4_ composites.

Sr. No.	Nanocomposites	Manganese Chloride	Zinc Chloride	Ferric Chloride	S-Doped g-C_3_N_4_
1	Mn-ZnFe_2_O_4_	0.185 g	1.706 g	2.435 g	-
2	S-g-C_3_N_4_	-	-	-	0.52 g
3	9% Mn-ZnFe_2_O_4_/10S-g-C_3_N_4_	0.185 g	1.706 g	2.435 g	0.17 g
4	9% Mn-ZnFe_2_O_4_/30S-g-C_3_N_4_	0.185 g	1.706 g	2.435 g	0.52 g
5	9% Mn-ZnFe_2_O_4_/50S-g-C_3_N_4_	0.185 g	1.706 g	2.435 g	0.87 g
6	9% Mn-ZnFe_2_O_4_/70S-g-C_3_N_4_	0.185 g	1.706 g	2.435 g	0.94 g

**Table 2:** The rate constant (k) values of the 9% Mn-ZnFe_2_O_4_/50% S-g-C_3_N_4_ nanocomposites.

Sr. No.	Nanocomposites	S-g-C_3_N_4_ (wt.%)	% Degradation	k (min^−1^)
1	S-g-C_3_N_4_	100	51	0.00515
2	ZnFe_2_O_4_	-	54	0.00673
3	9% Mn-ZnFe_2_O_4_/10%S-g-C_3_N_4_	10	65	0.00721
4	9% Mn-ZnFe_2_O_4_/30%S-g-C_3_N_4_	30	89	0.00838
5	9% Mn-ZnFe_2_O_4_/50% S-g-C_3_N_4_	50	100	0.0142
7	9% Mn-ZnFe_2_O_4_/70% S-g-C_3_N_4_	70	85	0.0096

**Table 3:** Comparison of photocatalytic efficiency of the Mn-ZnFe_2_O_4_/S-g-C_3_N_4_ NCs with some previous works.

Sr. No.	Photocatalyst	Contaminant	Light Source	Radiation Time (min.)	Degradation %	Ref.
1	ZnFe_2_O_4_@metyle cellulose	Metronidazole	Xe lamp	120	92.65	[49]
2	Bi_2_WO_6_/CoFe_2_O_4_	Bisphenol A	Solar	120	92	[50]
3	ZnNd_x_Fe_2−x_O_4_	Rhodamine B	Xe lamp	180	98	[51]
4	ZnFe_2_O_4_	Toluene	Xe lamp	300	57.2	[52]
5	ZnO/Fe_3_O_4_/g-C_3_N_4_	MO	Visible	150	97.87	[53]
5	Pt-BiFeO_3_	MG	Solar	240	96	[54]
7	Mn–ZnO/RGO	RhB	Visible	140	99	[55]
8	ZnFe_2_O_4_@ZnO	MO	Visible	240	240	[56]
9	Mn-ZnFe_2_O_4_/S-g-C_3_N_4_	MB	Solar	120	100	Present Work

## Data Availability

The datasets generated during and/or analyzed during the current study are available from the corresponding author upon reasonable request.

## Supplementary Materials

The following supporting information can be downloaded at: https://www.mdpi.com/article/10.3390/molecules27206925/s1.
