# Supplementary material for: Integration of Mn-ZnFe2O4 with S-g-C3N4 for Boosting Spatial Charge Generation and Separation as an Efficient Photocatalyst
Source: Molecules. 2022 Oct 15;27(20):6925. doi: 10.3390/molecules27206925 (PMC9610048; doi:10.3390/molecules27206925)
Supplement: Supplementary file 1 [file molecules-27-06925-s001.zip › molecules-1949428-supplementary.pdf]

Supporting Information

Integration of Mn-$ZnFe_2O_4$ with S-g-C_3N_4 for boosting spatial charge generation and separation as efficient photocatalyst

Mohsin Javed[1], Waleed Bin Khalid[1], Shahid Iqbal[2*], Muhammad Azam Qamar[1], Hamad Alrbyawi[3], Nasser S. Awwad[4], Hala A. Ibrahium[5,6], Murefah Mana Al-Anazy[7], Eslam B. Elkaeed[8], Rami Adel Pashameah[9], Eman Alzahrani[10], Abd-ElAziem Farouk[11]

[1]Department of Chemistry, School of Science, University of Management and Technology, Lahore 54770, Pakistan.

[2]Department of Chemistry, School of Natural Sciences (SNS), National University of Science and Technology (NUST), H-12, Islamabad, 46000, Pakistan.

[3]Pharmaceutics and Pharmaceutical Technology Department, College of Pharmacy, Taibah University, Medina Saudi Arabia.

[4]Chemistry Department, Faculty of Science, King Khalid University, P.O. Box 9004, Abha 61413, Saudi Arabia.

[5]Biology Department, Faculty of Science, King Khalid University, P.O. Box 9004, Abha 61413, Saudi Arabia.

[6]Department of Semi Pilot Plant, Nuclear Materials Authority, P.O. Box 530, El Maadi, Egypt.

[7]Department of Chemistry, College of Science, Princess Nourah bint Abdulrahman University, P.O. Box 84428, Riyadh 11671, Saudi Arabia.

[8]Department of Pharmaceutical Sciences, College of Pharmacy, AlMaarefa University, Riyadh 13713, Saudi Arabia.

[9]Department of Chemistry, Faculty of Applied Science, Umm Al-Qura University, Makkah 24230, Saudi Arabia.

[10]Department of Chemistry, College of Science, Taif University, P.O. Box 11099, Taif 21944, Saudi Arabia.

[11]Department of Biotechnology College of Science, Taif University, P.O. Box 11099, Taif 21944, Saudi Arabia.

To whom corresponding should be addressed

shahidgcs10@yahoo.com (Shahid Iqbal)

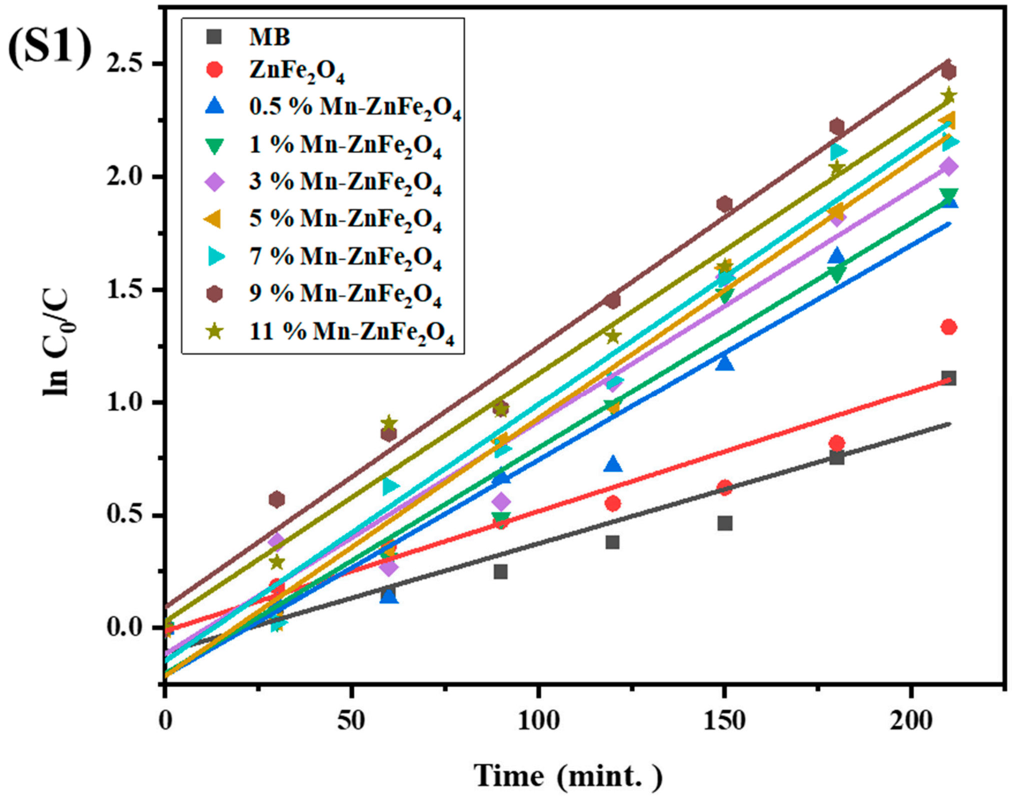

Figure S1 First-order kinetics plot of $ZnFe_2O_4$ and Mn-$ZnFe_2O_4$ NPs.

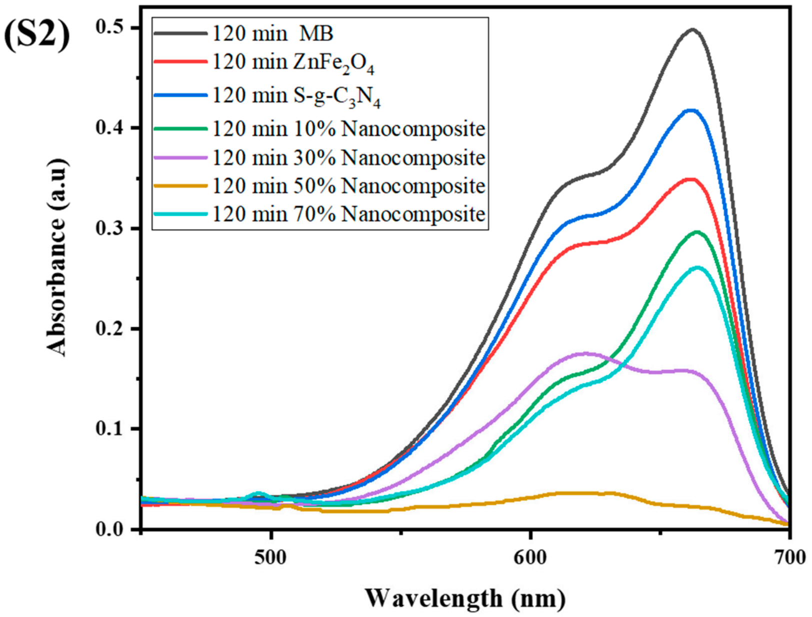

Figure S2 Photodegradation of MB by Mn-$ZnFe_2O_4$/S-g-C_3N_4 NCs after 120 minutes of sunlight irradiation (Degradation contours)

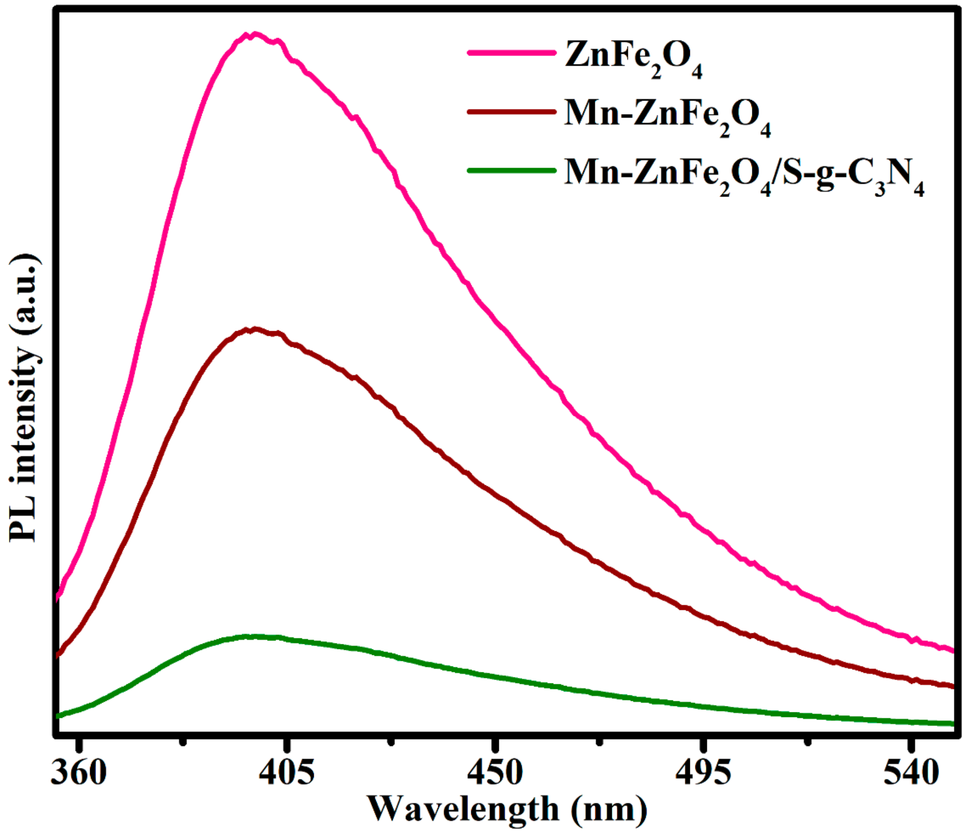

Figure S3. PL spectra of $ZnFe_2O_4$, Mn-$ZnFe_2O_4$ and Mn-$ZnFe_2O_4$/S-g-C_3N_4 heterostructures at an excitation wavelength of 330 nm.
